# Development of Microsatellite Markers for *Ex Situ* Management of the Harpy Eagle Using Next Generation Sequencing

**DOI:** 10.1002/zoo.70030

**Published:** 2025-10-09

**Authors:** Mylena Kaizer, Pedro S. Bittencourt, Érico M. Polo, Tânia M. Sanaiotti, Izeni P. Farias, Lorenzo von Fersen, Tomas Hrbek, Aureo Banhos

**Affiliations:** ^1^ Programa de Pós‐Graduação em Zoologia ‐ PPGZOOL Universidade Federal do Amazonas Manaus Amazonas Brazil; ^2^ Laboratório de Evolução e Genética Animal ‐ LEGAL, Departamento de Genética Universidade Federal do Amazonas Manaus Amazonas Brazil; ^3^ Projeto Harpia Instituto Nacional de Pesquisas da Amazônia ‐ INPA Manaus Amazonas Brazil; ^4^ Programa de Pós‐Graduação em Ciências Biológicas (Biologia Animal) ‐ PPGBAN Universidade Federal do Espírito Santo Vitória Espírito Santo Brazil; ^5^ Fundação Espírito‐Santense de Tecnologia ‐ FEST Vitória Espírito Santo Brazil; ^6^ Coordenação de Biodiversidade Instituto Nacional de Pesquisas da Amazônia ‐ INPA Manaus Amazonas Brazil; ^7^ Nuremberg Zoo Nuernberg Germany; ^8^ Biology Department Trinity University San Antonio TX USA; ^9^ Departamento de Biologia, Centro de Ciências Exatas, Naturais e da Saúde ‐ CCENS Universidade Federal do Espírito Santo Alegre Espírito Santo Brazil

**Keywords:** Accipitridae, *ex situ* conservation, *Harpia harpyja*, relatedness

## Abstract

The Harpy Eagle (*Harpia harpyja*), one of the largest birds of prey in the world, is threatened with extinction throughout its entire area of occurrence in the Neotropics. While conservation efforts for the wild populations are crucial, it is also important to focus on *ex situ* conservation actions. To achieve this, understanding the genetic characteristics of the captive population is essential to prevent inbreeding and loss of genetic diversity over time. In this study, we employed a next generation sequencing strategy to develop a new set of primers for microsatellite regions specific to the Harpy Eagle. These markers were validated in Harpy Eagle individuals maintained in captivity at Brazilian zoos and conservation institutions. We characterized these captive individuals using ten highly polymorphic microsatellite loci to estimate relatedness and individual identification at > 95% accuracy. The same effect can be achieved with four loci. Additionally, we developed a statistical test to further refine relationship estimates. Paired with robust sex markers, the new set of microsatellite markers presented here the potential to guide *ex situ* management strategies, aiming for better reproductive pairings and the maintenance of genetic diversity of the Harpy Eagle.

## Summary

- The new microsatellite markers developed for Harpy Eagle in this study are efficient for (1) quantifying relatedness between individuals; (2) performing identification of individuals and (3) excluding relatives.
- The genetic information of the individuals can be entered into their record to assist zoos and breeding grounds dedicated to the conservation of the Harpy Eagle carry out the *ex situ* management in an assertive way.
- The Next Generation genotyping protocol using the Ion Torrent PGM platform was efficient. It accelerated the process of obtaining results, which will be used to make decisions regarding the *ex situ* management of the Harpy Eagle.

## Graphical abstract

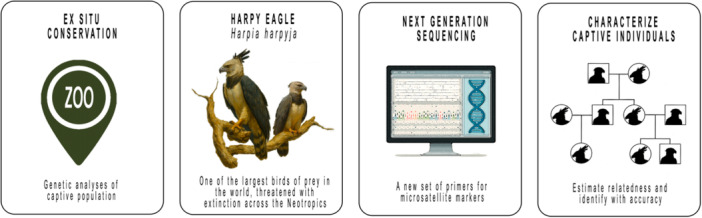

## 1 Introduction

*Harpia harpyja* (Linnaeus, 1758), commonly known as the Harpy Eagle, belongs to the family Accipitridae and is recognized as one of the largest living eagles in the world (Sick 1997). Its habitat consists of dense tropical and gallery forests in Central and South America (Rudnick and Lacy 2008; Vargas et al. 2006). Unfortunately, the Harpy Eagle has experienced significant population declines across its range, primarily due to habitat loss and hunting (Banhos et al. 2018; Trinca et al. 2008). The IUCN estimates that the Harpy Eagle population may decline by up to 50% over the next three generations (BirdLife International 2021). Consequently, the IUCN has categorized the species as Vulnerable (BirdLife International 2021).

Both *in situ* and *ex situ* conservation programs have been implemented to reduce the threat of extinction of the Harpy Eagle. While *in situ* actions focus on protecting the species′ nests and their immediate area of use (Aguiar‐Silva et al. 2015; Banhos et al. 2023; Kaizer et al. 2023; Sanaiotti et al. 2015), *ex situ* actions primarily focus on the rescuing, rehabilitating and releasing injured individuals (Francisco and Silveira 2013; Oliveira et al. 2022). Individuals that cannot be reintroduced into the wild are held in zoos or with breeders dedicated to the species′ conservation. These animals, along with the institutions that house them, play a crucial role in the *ex situ* conservation of the Harpy Eagle, as they participate in a captive breeding program. The captive bred individuals can, through the release of individuals, be used to supplement existing wild populations or even establish new ones in the wild (Frankham et al. 2017; IUCN/SSC 2014; Soares et al. 2008).

Harpy Eagles have been reproducing successfully in captivity in Brazil since 1999 (Azeredo 2005). However, the selection of breeding pairs has been carried out without the use of molecular information, relying solely on behavioral compatibility between individuals and data about individuals such as age, origin, and sex (Oliveira et al. 2022; Watson et al. 2016). Moreover, incorrect or incomplete information about individuals is common. This can lead to an underestimation of the degree of relatedness between breeders (Bowling et al. 2003; Modesto et al. 2018; Gonçalves da Silva et al. 2010) and to incorrect sex identification. The Harpy Eagle exhibits no sexual dimorphism except that females tend to be larger than males. This has led to the formation of same‐sex pairs in captive breeding programs. The use of molecular tools can help mitigate these issues since individual genotypes can be used to estimate relatedness between individuals, the geographic origin of these individuals, their genetic diversity and their sex.

Microsatellites (Simple Sequence Repeats, SSRs) are codominant, hypervariable molecular markers that allow the determination of genetic identity and the assessment of relatedness among individuals (Turchetto‐Zolet et al. 2017). Due to these properties, many *ex situ* conservation programs make use of microsatellites to perform detailed analyses of relatedness and genetic diversity (Aliaga‐Samanez et al. 2023; Ayala‐Burbano et al. 2017; Campos et al. 2021; Mukesh et al. 2016; Henkel et al. 2012; Kleinman‐Ruiz et al. 2019; Witzenberger and Hochkirch 2011, 2013). The information derived from these analyses is then used to minimize inbreeding and maximize genetic diversity of the *ex situ* population.

Previous studies have used microsatellites to assess the genetic diversity of the Harpy Eagle (Banhos et al. 2008). However, the markers used were not specifically developed for this species; instead, they were transferred from other species of the order Falconiformes and Accipitriformes (Banhos et al. 2008). These microsatellites, known as heterologous markers, are a useful alternative when species‐specific markers are unavailable. However, their effectiveness depends on the genetic divergence of the species. If the genetic divergence is substantial, these markers will underestimate the true genetic diversity of the target species and reduce the accuracy of relatedness analyses (Barbará et al. 2007; Turchetto‐Zolet et al. 2017).

The advancement of high‐throughput sequencing platforms and new bioinformatic pipelines has paved the way for more advanced and cost‐effective techniques in microsatellite development (Gardner et al. 2011; Meglécz et al. 2014) and genotyping (Hoogenboom et al. 2017). This, in turn, enhances the efficiency of genetic analyses and accelerates decision‐making processes. In this context, we developed novel microsatellite loci specifically for the Harpy Eagle to create a molecular toolbox for assessing genetic diversity, relatedness, and sex determination in captive individuals, thereby supporting the *ex situ* management of this species.

## 2 Materials and Methods

### 2.1 Development of New Microsatellites

To isolate and characterize the new microsatellites specific to the Harpy Eagle, we designed primers from a genomic library generated on the Ion Torrent PGM sequencer. The library was created using a blood sample taken from an individual rescued by the Harpy Project (www.projetoharpia.org) in Parintins, Amazonas, Brazil (SRS18688466/SAMN31530492).

We used the ddRADseq methodology (Peterson et al. 2012) with some adaptations (Hrbek et al. submeted) to obtain a reduced representation of the genome. The principal steps in the preparation and sequencing of the genomic library are: (1) DNA extraction using 2% CTAB with addition of 15 mg/mL of proteinase K; (2) digestion of genomic DNA (200 ng) with the PstI (rare‐cutter; ER0611 ‐ Thermo Fisher Scientific) and Csp6I (frequent‐cutter; ER0211 ‐ Thermo Fisher Scientific) restriction enzymes, and simultaneous ligation (EL0021 ‐ Thermo Fisher Scientific) of the adapters P1 (rare cut) and A (frequent cut); (3) enrichment of the digestion/ligation product by polymerase chain reaction (PCR); (4) purification of the enriched product with SeraMag beads prepared using the BOMB #4.2 protocol (Oberacker et al. 2019); (5) size selection of purified sample fragments between 374 and 456 base pairs (bp) on Pippin Prep (Sage Science); (6) purification with beads and quantification in the Qubit 2.0 fluorometer (Thermo Fisher Scientific); (7) clonal emulsion PCR and loading of the 318 Ion PGM chip (Thermo Fisher Scientific) using the Ion Chef and (8) sequencing the fragments loaded onto the 318 Ion PGM chip in the Ion Torrent PGM using the Ion PGM Sequencing 400 kit (Thermo Fisher Scientific).

We converted the BAM output file to FASTQ format using the bam2fq function of the Samtools 1.13 (Danecek et al. 2021) and then filtered the FASTQ file to remove low quality ( > 5 sites with < Q20) and short ( ≤ 100 bp) reads. We then filtered and selected microsatellite containing sequences using the QDD3 pipeline (Meglécz et al. 2014) implementing the following parameters: (1) microsatellites containing at least 10 repeats (di‐ or trinucleotide motif) in length; (2) simple and perfect repeats; (3) PCR product size between 120 and 300 bp; (4) primer no less than 20 bp from the target microsatellite region; (5) primer length between 18 and 27 bp and annealing temperature between 57°C and 63°C; (6) consensus sequences with polymorphism detected in their reads and (7) implementation of “best primer design” (Meglécz et al. 2014) when multiple pairs of primers are suggested to amplify the same repetitive region, avoiding homopolymers, other repetitive regions and nanosatellites in the flanking region or in the primer sequence.

The forward and reverse primers of each microsatellite were tailed on their 5′ end with 5′‐CGCTCTTCCGATCT‐3′ and 5′‐AAACGACGGCCAGT‐3′, respectively. These tails are complementary to the 3′ ends of the P1 and A adapters of the Ion Torrent platform, and are used for generating fusion sequencing primers. To individualize samples, both adapters additionally contained a unique six nucleotide region index region which was used for demultiplexing individuals. We developed 19 primer pairs which were initially tested on eight individuals.

### 2.2 Sampling, DNA Extraction and Sex Identification

We received samples of 25 individuals of Harpy Eagle that were held in zoos and conservation institutions (Supporting Table 1). Of these, 23 individuals were born in the wild and two in captivity at the Itaipu Binacional Wild Animal Breeding Area (CASIB) belonging to the Bela Vista Biological Refuge (RBV; Supporting Table 1). Of the 23 wild‐born individuals, 13 came from the Amazon Rainforest, three from the Atlantic Forest, one from the central region of Brazil and six had unknown origin. Out of these six individuals with no known origin, three were likely from the state of Pará (Supporting Table 1).

We extracted the DNA from blood samples from 25 individuals of Harpy Eagle using a Qiagen blood and tissue kit, following the protocol provided by the manufacturer (Valencia, CA, USA). We assessed DNA quality by measuring DNA purity and concentration on the NanoDrop 2000 spectrophotometer (Thermo Fisher Scientific). We diluted all samples to a concentration of 20 ng/µL to standardize future reactions for all of them. We identified the sex of each individual using the molecular protocol developed by Banhos et al. (2008) (Supporting Information 1), and to assess the consistency of the results, we repeated the molecular sexing of all samples three times.

### 2.3 Sequencing Library Preparation

We initially amplified all 19 microsatellites individually in the eight Harpy Eagle samples to test the effectiveness of the new primers and to prevent the formation of chimeras. We evaluated all PCR products and selected 17 microsatellites that resulted in a single band of expected size in at least seven samples. Subsequently, we performed amplification of the 25 samples using the 17 chosen primers. Each PCR contained a final volume of 10 µL, consisting of: 1 µL of DNA, 3.2 µL of nuclease‐free ddH_2_O, 1 µL of dNTPs (1 mM), 1 µL of MgCl_2_ (2.5 mM), 1 µL of 10× PCR buffer (75 mM Tris‐HCl pH 8.8 at 25°C, 20 mM (NH_4_)_2_SO_4_), 0.5 µL BSA (2 mg/mL), 1 µL (2 mM) of each primer, forward and reverse and 0.3 µL of Taq DNA polymerase (0.1 U). PCR cycling conditions were: (1) initial denaturation at 94°C for 60 s; (2) 30 cycles of denaturation at 94°C for 30 s, primer annealing at a specific temperature for each primer for 30 s and extension at 68°C for 40 s; and (3) a final extension of 72°C for 300 s (Supporting Information 2).

We collected 3 μL of each PCR product per individual and purified it using SeraMag beads to remove fragments smaller than 100 bp using a ratio of 0.8 μL of beads per 1 μL of the pooled PCR product per individual (Supporting Information 2). The purified PCR products were resuspended in 10 μL of ddH_2_O. We used this pool to add P1 and A sequencing adapters to the samples, ensuring that each individual had a unique P1 index. For this PCR, we prepared the reaction mix separately for each adapter, containing: 1.8 μL of nuclease‐free ddH_2_O, 0.5 µL of dNTPs (1 mM), 0.5 µL of MgCl_2_ (2.5 mM), 0.5 µL of 10× PCR buffer (75 mM Tris‐HCl, pH 8.8 at 25°C, 20 mM (NH_4_)_2_SO_4_), 0.25 µL of BSA (2 mg/mL), 0.4 µL of primer A or P1 (0.2 pM), and 0.15 µL of Taq DNA polymerase (0.1 U). Afterward, 4.5 μL of each adapter (P1 and A) was mixed with 1 μL of the purified PCR product, resulting in a final volume of 10 µL. The PCR cycling conditions for adapter ligation were: (1) initial denaturation at 94°C for 60 s; (2) 15 cycles of denaturation at 94°C for 30 s, adapter annealing at 48°C for 30 s, and extension at 68°C for 40 s; (3) final extension at 72°C for 420 s (Supporting Information 2).

**Figure 1:**
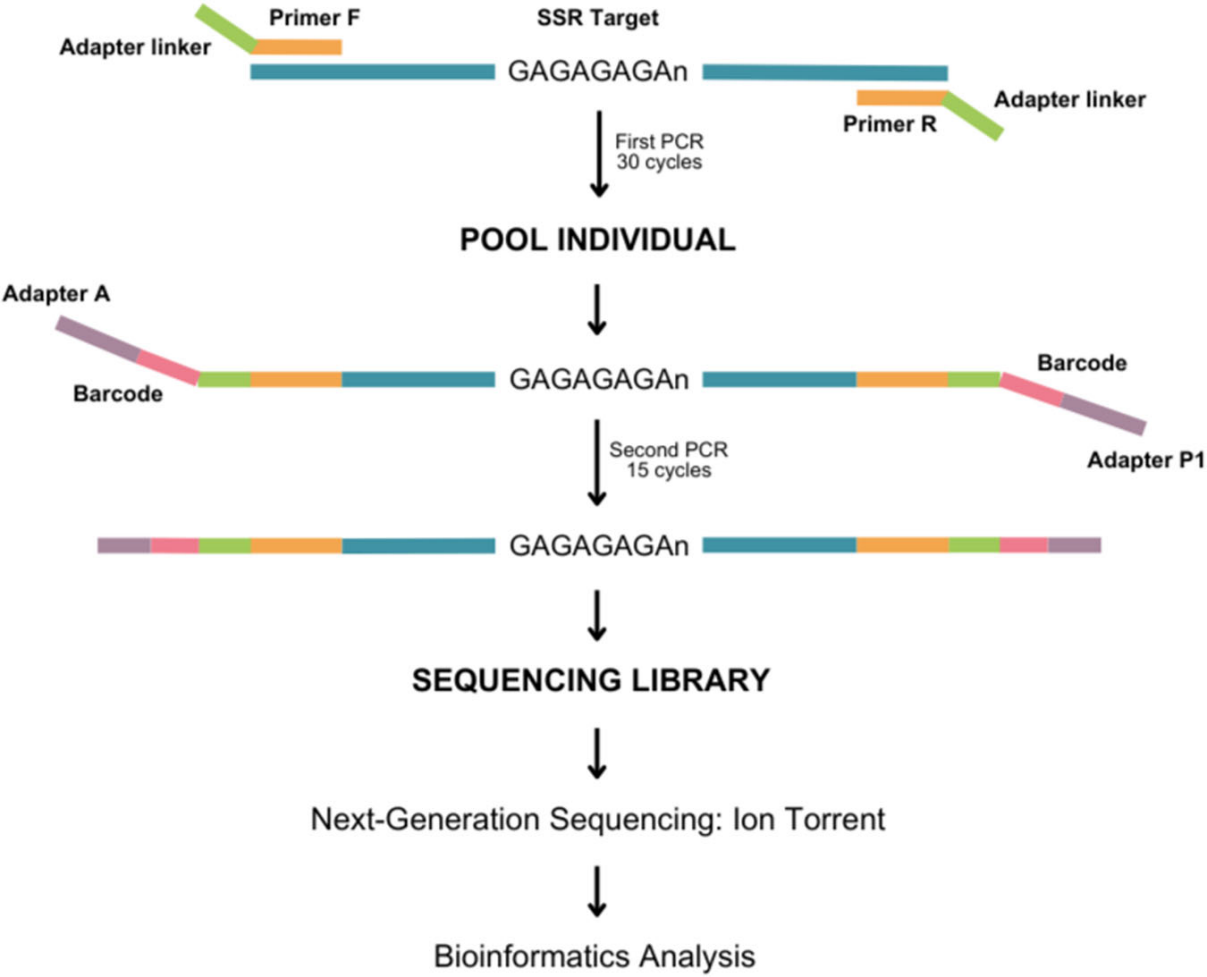
Schematic representation of the amplification strategy and library preparation for next generation sequencing using the Ion Torrent platform.

We confirmed the incorporation of the indexed adapters on a 1% agarose gel, and pooled the individual PCR products by taking 3 μL from each pool (Supporting Information 2). The combined pool, containing all individuals was re‐purified using SeraMag beads and eluted in nuclease‐free ddH_2_O under the same conditions as the previous purification step, generating a sequencing library. The library was then subjected to emulsion PCR and loaded onto the Ion PGM 318 chip (Thermo Fisher Scientific) using the Ion Chef (Thermo Fisher Scientific). Sequencing was then performed on the Ion Torrent PGM (Thermo Fisher Scientific), following the manufacturer′s instructions for the Ion PGM 318 chip and Ion PGM Sequencing 400 kit chemistry (Figure 1).

Afterward, we converted the resulting BAM output from the sequencing library to FASTQ format and used fqgrep (github.com/indraniel/fqgrep) to demultiplex the reads by individual (unique combination of P1 and A indexes). Subsequently, we demultiplexed each individual by microsatellite pairs (forward and reverse primers). Each resulting FASTQ file contained reads specific to a particular microsatellite for a given individual.

### 2.4 Genotyping and Statistical Analysis

We analyzed each FASTQ file using FDSTools (Hoogenboom et al. 2017). For each individual, we extracted the full sequence, including the microsatellite region and its flanking regions. A FASTA file was generated for each locus, containing the allelic variations of all individuals, as identified by the FDSTools allelefinder tool. Lastly, we manually aligned and edited the sequences using AliView (Larsson 2014).

We generated four genotyping databases, namely: (1) FullLength – the full length of the sequence, along with the flanking regions and the microsatellite region; (2) RepeatFocused – target microsatellite region size only; (3) SSRs – the size of the target microsatellite region and any other types of repeats observed in the complete sequence and (4) CAT – concatenated data, that is, all types of polymorphism found in the sequence (repeats + SNPs + indels) in a categorical way. We filtered all datasets using a custom R script (R Core Team 2022), selecting only samples and loci with at most 10% missing data. Although FDSTools (Hoogenboom et al. 2017), already automatically removes artifacts such as PCR stutter, null alleles, and other types of systemic noise, we performed an additional check for these artifacts in the four datasets using Micro‐Checker v2.2.3 (Van‐Oosterhout et al. 2004).

For genetic diversity analyses, we excluded captive‐born Harpy Eagles (HZ15 and HZ16) because their parents were also sampled in this study. We calculated genetic diversity parameters, including the number of alleles (N_A_), observed heterozygosity (H_O_), expected heterozygosity (H_E_), and Hardy–Weinberg equilibrium (HWE), using Arlequin 3.5 (Excoffier and Lischer 2010). Additionally, we tested the statistical significance of linkage disequilibrium between loci using FSTAT 2.9.4 (Goudet 1995).

We calculated the probability of paternity exclusion (Q), the joint probability of paternity exclusion across all loci (QC), the probability of genetic identity (I), and the joint probability of genetic identity across all loci (IC) in GenAlEx 6.5 (Peakall and Smouse 2012). The probability of paternity exclusion indicates the efficiency of a locus in excluding a random individual in the sample as a potential parent; thus, the closer the probability is to 1, the more informative the locus becomes. The probability of genetic identity is the probability of two individuals drawn at random from the sample having an identical genotype; thus, the closer the probability is to 0, the more informative the locus becomes.

To investigate the power of the microsatellite markers to infer relationships of individuals, we analyzed our data in ML‐Relate (Kalinowski et al. 2006). The program calculates pairwise relationship coefficients (*r*) based on maximum likelihood estimates, and classifies them into the four most common types of relatedness (*R*): unrelated (U), half‐siblings (HS), full‐siblings (FS) and parent‐offspring (PO). Since classification of relationships is probabilistic, we also ran 100,000 simulations to generate a 95% confidence interval of relationships. Using the results of the simulations, we adopted a conservative approach to relatedness classification. We considered individuals to be related only if the 95% confidence interval did not include the unrelated category. Conversely, we considered individuals to be unrelated only if the 95% confidence interval did not include any of the related categories. Pairs of individuals whose 95% confidence interval included both unrelated and related relationship categories were classified as having an ambiguous relationship.

All ambiguous pairs underwent hypothesis testing to statistically confirm the most probable relationship. To do this, we use a likelihood ratio test between the relationship with the highest probability and the alternative relationships estimated by the confidence interval. For each test, 100,000 simulations were performed, and alternative relationship classifications were rejected in favor of the most likely one, based on a significance threshold. To correct for multiple testing, *p*‐values were adjusted using the Bonferroni correction, causing the significance threshold to vary according to the total number of tests performed.

To verify the effectiveness of the new microsatellite markers in determining kinship between individuals, we included in our data set a family of Harpy Eagles comprised of a wild‐born female (HZ12), a wild‐born male (HZ13) and their two offspring (HZ15 and HZ16).

## 3 Results

### 3.1 Development of New Microsatellites

Our filtered Harpy Eagle genomic library consisted of 141,281 reads ranging from 101 to 605 bp in length, with an average size of 311 bp. These reads were processed using the QDD3 pipeline. Of the 141,281 reads, 2860 reads presented microsatellite regions (PIPE1 of QDD3) of which 1046 were singletons and 412 unique consensus sequences (PIPE2 of QDD3). Of these 1458 sequences, 1020 sequences contained microsatellites and flanking regions suitable for primer design. In the 1020 microsatellite sequences, the most frequent repeat pattern was dinucleotides (78.52%), followed by tri (16.67%), tetra (3.13%), penta (1.18%) and hexa (0.50%). Of these available microsatellites, we chose the top 19 (15 dinucleotide and 4 trinucleotide) following our selection criteria. The 19 primer pairs were tested for PCR amplification efficiency in eight Harpy Eagle samples, and of these 17 primer pairs were selected for genotyping of all samples since they amplified at least seven of the eight samples.

### 3.2 Sampling and Sex Identification

The institutions that provided us with the samples also provided us with information about individuals, including the sex. In principle, these samples consisted of 12 males and 13 females. After determining the sex through DNA analysis, we confirmed that all previous identifications were correct.

### 3.3 Characterization of New Microsatellites

After demultiplexing our genotyping library, we retained 306,403 reads. The number of reads per individual ranged from 8859 to 15,793 with an average of 12,256 reads per individual. We subsequently extracted the 17 microsatellites for all individuals. Locus Hha16 had a very low read count, while four other loci (Hha07, Hha11, Hha13, and Hha15) exhibited more than 10% missing data. Consequently, we excluded these loci from the analysis. Further two loci (Hha10 and Hha17) were monomorphic and were also removed. One individual (HZ14) had excessive missing data and needed to be excluded from the final data set (Supporting Table 2). Consequently, our data set included 10 loci and 24 individuals for microsatellite characterization (Table 1; Supporting Table 2).

We used the selected loci and samples to generate four different types of datasets, as described in the Methods section (FullLength, RepeatFocused, SSRs, and CAT; see the Genotyping and Statistical Analysis section). The results for genetic diversity, individual identification, and kinship exclusion were consistent across all four datasets. Therefore, we chose to use the FullLength data set for all subsequent analyses, as it was the easiest to extract from the genomic reads and is conceptually identical to data obtained through traditional genotyping methods (fragment length analysis using Sanger sequencing), making the microsatellite markers developed here well‐suited for standard fragment length analyses.

The 10 microsatellite loci showed no stutter artifacts, null alleles, or linkage disequilibrium after applying Bonferroni correction. However, four loci (Hha02, Hha03, Hha06, and Hha09) exhibited significant deviations (*p* < 0.05) from Hardy–Weinberg equilibrium (HWE). The number of alleles per locus (N_A_) ranged from 2 to 8, with an average of 4.4 alleles per locus. Observed (H_O_) and expected (H_E_) heterozygosities ranged from 0.0454 to 0.8636 and 0.0454 to 0.8266, respectively, with average values of 0.3636 for H_O_ and 0.4534 for H_E_ (Table 1). The joint probability of paternity exclusion across all loci (QC) was 0.9946, while the joint probability of genetic identity (IC) across all loci was 2.0362×10^−5^ (Table 1; Supporting Table 3). Interestingly, a subset of just four loci (Hha02, Hha03, Hha05, and Hha06) exhibited the same discriminatory power as the full set of 10 loci, with QC = 0.9696 and IC = 1.0204×10^−3^ (Supporting Table 3).

**Table 1:** Characterization of 10 microsatellite loci in 24 individuals of Harpy Eagle (*Harpia harpyja*) held in captivity in Brazil.

Locus	Repetition Motif	Primer Sequence (5′ − 3′)	Size (bp)	T_a_ (°C)	N	N_A_	H_O_	H_E_	P_HWE_	Q	I
Hha01	(AC)_**10**_	F‐ CTCCTGTGTTCCCATCTGCT	158	59	24	2	0.2727	0.2410	1.000	0.1759	0.6121
		R‐ GGGTGCGAGACAGTTCCTC									
Hha02	(AC)_**10**_	F‐ TCTGTTGGAGTTTCCGGAGC	192	59	24	8	0.5000	0.8266	0.000*	0.7517	0.0837
		R‐ CCGGGATGCATCTCCTTTGT									
Hha03	(AG)_**14**_	F‐ ATGGCAAGTCGCTAGAACGG	204	57	24	5	0.5454	0.6797	0.003*	0.5037	0.2176
		R‐ GGCTGCTATTCCACTTGCCA									
Hha04	(AC)_**12**_	F‐ GTGGAAGGGAGTTAGCGTGG	125	59	24	2	0.0454	0.0454	1.000	0.0420	0.9141
		R‐ ATGGCTCTGTCTAACCTGCG									
Hha05	(AGC)_**11**_	F‐ ACAAGGTCACATCTTCCCGC	179	59	24	8	0.5000	0.5549	0.231	0.4602	0.3149
		R‐ AAACTGTTCTGCCTCGCTGA									
Hha06	(AG)_**12**_	F‐ ACCAGGCTGTAAGGGTTGAAG	121	59	24	4	0.8636	0.6839	0.000*	0.5438	0.1777
		R‐ TGGACAGATGTGGAGTGTCG									
Hha08	(AC)_**10**_	F‐ CATCCGTAGCCATGCACAGA	155	57	24	4	0.2727	0.3541	0.218	0.3067	0.4545
		R‐ CTTACCTGGGTGTCAGCACA									
Hha09	(AC)_**12**_	F‐ ACCCAAGCTCTTCCTGGATG	198	57	24	4	0.0909	0.4904	0.000*	0.3990	0.3233
		R‐ TCAAAGCGTATGAAGCCAGGA									
Hha12	(AG)_**11**_	F‐ TCCTCCTTGGAAGCAGCAAG	120	57	24	3	0.3181	0.3689	0.225	0.2654	0.4595
		R‐ AGCTCAGTTTCTGGAGTCAGG									
Hha18	(AC)_**11**_	F‐ GCCCACAGAAACGATACAGC	150	57	24	4	0.2272	0.2896	0.265	0.2684	0.5279
		R‐ ATTAGCTGCTCGCAGACAAA					average *=* 0.3636	average *=* 0.4534		QC = 0.9946	IC = 2.0362×10^–5^

Abbreviations: bp, base pairs; F, forward; H_E_, expected heterozygosity; H_O_, observed heterozygosity; I, probability of genetic identity at a locus; IC, probability of genetic identity for all loci; N, number of genotyped individuals; N_A_, number of alleles; P_HWE_, probability of deviation from Hardy‐Weinberg equilibrium (*p* value); Q, probability of paternity exclusion at a locus; QC, paternity exclusion probability for all loci; R, reverse; T_a_, primer pair annealing temperature (°C). Significant deviation from Hardy–Weinberg equilibrium at

* *p* < 0.05.

**Figure 2:**
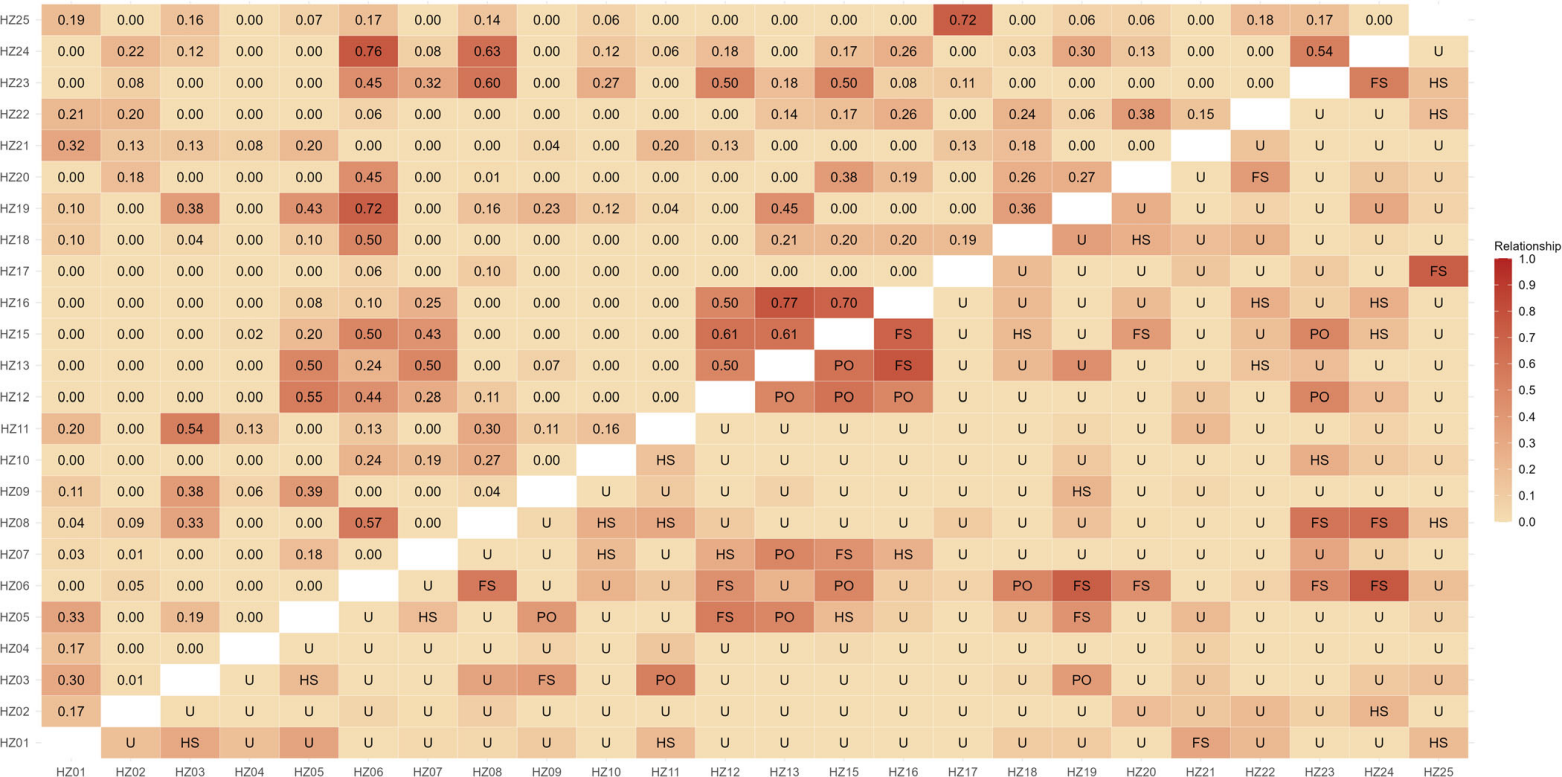
Heatmap of pairwise relationship coefficients (*r*) and their respective highest‐likelihood relationships among captive Harpy Eagle individuals sampled in this study. Types of relatedness (*R*): unrelated (U), half‐siblings (HS), full‐siblings (FS) and parent‐offspring (PO).

By analyzing the most likely pairwise relationship coefficients (*r*) and their respective categories, we identified 220 pairs classified as unrelated (U) and 56 pairs with some degree of relatedness. Among these, 24 were categorized as half‐siblings (HS), 19 as full‐siblings (FS), and 13 as parent‐offspring pairs (PO) (Figure 2; Supporting Table 4). However, only 19 pairs were definitively classified as related (Table 2; Supporting Table 5), while 49 were confirmed as unrelated (Supporting Table 5). The remaining 208 pairs exhibited ambiguous relationships, as they included both the unrelated (U) category and some degree of relatedness (HS, FS, and/or PO) within the confidence interval (Supporting Table 5). Among the 19 unequivocally related pairs, 13 were FS and six were PO (Table 2).

**Table 2:** Maximum likelihood estimates of relationship for Harpy Eagles with unambiguous relationships based on ML‐Relate.

Individual 1	Individual 2	*r*	*R*	95% IC
HZ01	HZ21	0.3171	FS	FS
HZ03	HZ09	0.3786	FS	HS, FS
HZ03	HZ11	0.5397	PO	FS, PO
HZ05	HZ12	0.5486	FS	HS, FS
HZ05	HZ13	0.5000	PO	HS, FS, PO
HZ05	HZ19	0.4331	FS	FS
HZ06	HZ08	0.5684	FS	HS, FS, PO
HZ06	HZ19	0.7178	FS	HS, FS
HZ06	HZ24	0.7607	FS	FS
HZ07	HZ13	0.5000	PO	HS, FS, PO
HZ08	HZ23	0.6045	FS	FS, PO
HZ08	HZ24	0.6260	FS	FS
HZ12	HZ15	0.6075	PO	HS, FS, PO
HZ12	HZ16	0.5000	PO	HS, FS, PO
HZ13	HZ15	0.6138	PO	HS, FS, PO
HZ13	HZ16	0.7651	FS	FS
HZ15	HZ16	0.7025	FS	HS, FS, PO
HZ17	HZ25	0.7176	FS	FS, PO
HZ23	HZ24	0.5408	FS	HS, FS, PO

Abbreviations: *r*, maximum likelihood estimates of relatedness; *R*, relationship with the highest likelihood; 95% IC, shows which relationships are consistent with the genetic data at the 0.05 level of significance. Types of relatedness (R): unrelated (U), half‐siblings (HS), full‐siblings (FS) and parent‐offspring (PO).

**Figure 3:**
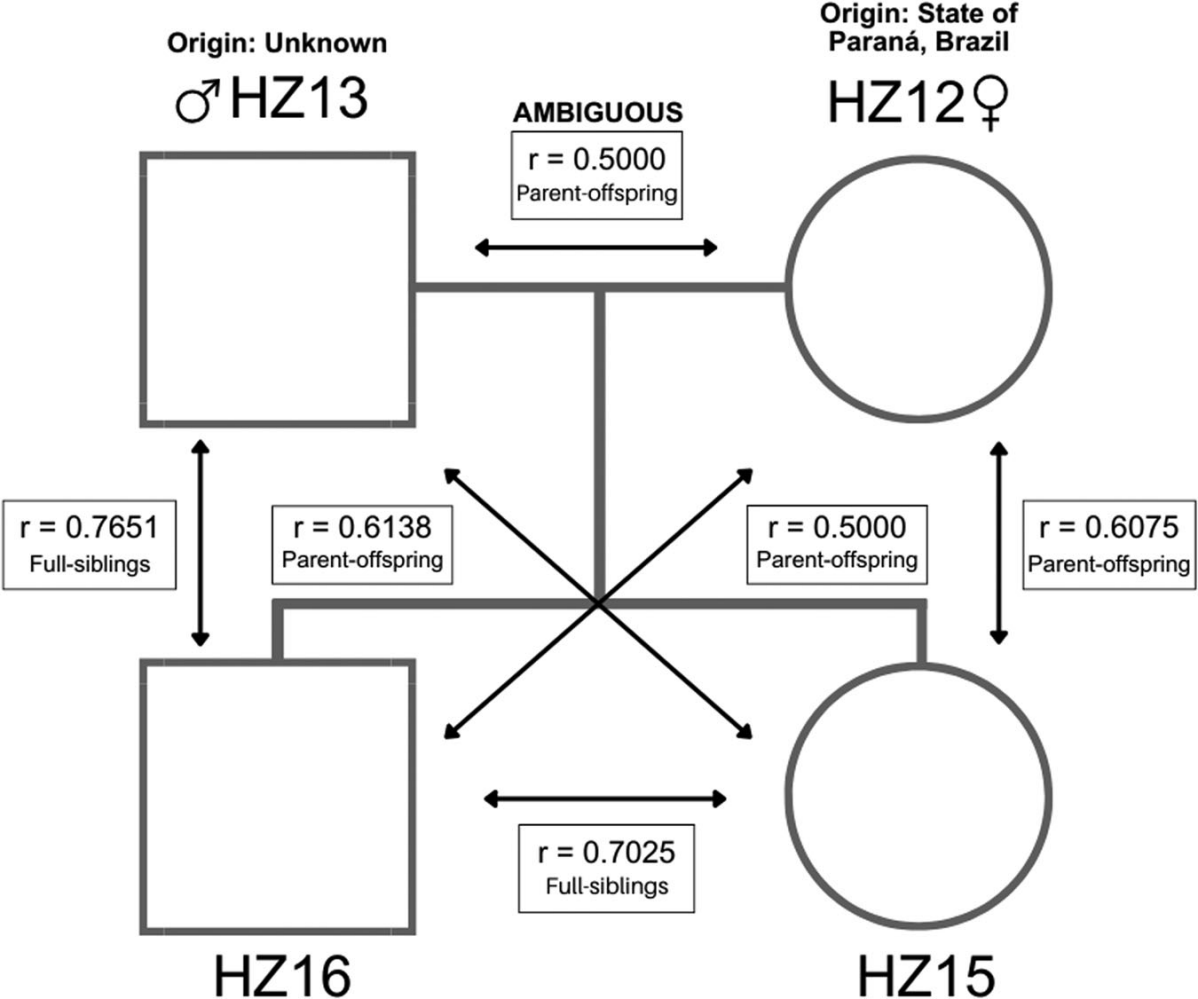
Pedigree showing the pairwise relatedness coefficients (*r*) in a captive Harpy Eagle family.

This group of unambiguously related individuals includes a family of four members. HZ15 and HZ16, who are full‐siblings, were born at CASIB from a cross between HZ12, a female of unknown origin, and HZ13, a male from Paraná, Brazil, near the border with Argentina, both born in the wild (Figure 3; Supporting Table 1). The relationships within this family were accurately identified, with high relationship coefficients ranging from 0.5 to 0.7651 (Table 2). The only exception was the parent‐offspring relationship of HZ13 and HZ16, which had a coefficient of *r* = 0.7651 but was classified as full‐siblings (FS) instead of parent‐offspring (PO) (Table 2; Figure 3; Supporting Tables 4 and 5).

Excluding the family with a previously known relationship, 14 related pairs remained (Table 2). Of these, six cases consisted of individuals from nearby localities in the state of Pará (HZ03‐HZ09, HZ06‐HZ08, HZ06‐HZ24, HZ08‐HZ23, HZ08‐HZ24 and HZ23‐HZ24). In one case, the individuals were from neighboring states, one from Pará and Amapá (HZ17‐HZ25). In four other cases, the individuals were from more distant localities, originating in the states of Pará and Bahia (HZ05‐HZ19 and HZ06‐HZ19) and of Pará and Paraná (HZ05‐HZ13 and HZ07‐HZ13). The remaining three cases involved pairs of individuals where one individual had a known origin and the other had an unknown origin. One case involved the state of Rondônia (HZ01‐HZ21) and two cases involved the state of Pará (HZ03‐HZ11 and HZ05‐HZ12) (Table 2; Supporting Table 1).

Hypothesis tests were conducted for the 208 pairs classified as ambiguous. Among them, 37 showed evidence of relatedness but remained ambiguous due to the inclusion of the U category in their confidence intervals (Supporting Table 5). Even after testing, the U category could not be ruled out for any of these pairs, maintaining the uncertainty regarding the type of relationship. Conversely, among the 171 cases initially classified as unrelated but exhibiting some degree of relatedness within their confidence intervals, we resolved approximately 41% (*N* = 70) of the ambiguities, confirming the absence of relatedness (Supporting Table 5). However, 101 cases, initially unrelated, remained ambiguous, indicating that these pairs could either be unrelated or share some level of relatedness, such as HS, FS or PO. In total, after all hypothesis testing, 138 pairs remained ambiguous (Supporting Table 5).

## 4 Discussion

In this study, we successfully developed and characterized a new set of microsatellite markers specific to the Harpy Eagle, with the aim of obtaining detailed genetic information about the individuals and the population held in captivity. These markers allow for the evaluation of genetic diversity within any population, individual identification, and the calculation of pairwise relationship coefficients, facilitating the exclusion of kinship.

The microsatellites developed for the Harpy Eagle in this study showed lower diversity levels when compared to the study carried out using heterologous microsatellites by Banhos et al. (2016). This result is the same as that observed by Coser et al. (2014) who also developed Harpy Eagle specific microsatellites and found low levels of genetic diversity for the species (H_O_ = 0.364 and H_E_ = 0.365), suggesting that it is a specific characteristic of microsatellite markers for this species. The study of Coser et al. (2014), Banhos et al. (2016) and this study found lower diversity levels in the Harpy Eagle when compared to other eagles also classified as vulnerable, such as *Aquila adalberti* (H_O_ = 0.516 and H_E_ = 0.549) and *Aquila heliaca* (H_O_ = 0.563 and H_E_ = 0.627) (Martínez‐Cruz et al. 2004). Additionally, Banhos et al. (2016) detected a temporal loss of genetic diversity in the Harpy Eagle sampled in the Amazon Rainforest and Atlantic Forest.

The low genetic diversity found in microsatellites is worrying for the long‐term viability of the Harpy Eagle, considering the important role of genetic diversity in permitting species to adapt to environmental changes (Frankham et al. 2010). While our sample sizes are smaller than that of Banhos (2009) and Banhos et al. (2016) and do not represent the total geographic extent of the species, diversity is clearly low. This inference is supported by the analysis of the complete genome of the Harpy Eagle which also indicated drastic demographic decline (Canesin et al. 2024). These findings highlight the importance of monitoring the genetic diversity of the Harpy Eagle and implementing appropriate management strategies to ensure its maintenance or even maximize long‐term diversity. Genetic diversity needs to be accounted for when proposing and implementing conservation strategies to maximize the success of these conservation strategies (O'Brien et al. 2025).

The four microsatellite loci which showed deviation from HWE (Table 1) could be due to the Wahlund effect which becomes increasingly more pronounced as the differentiation among populations increases (Waples 2015). Previous studies have shown that the Harpy Eagle is genetically structured across Brazilian biomes (Banhos 2009; Gusmão et al. 2016). This information also needs to be considered in *ex situ* management. Although mating individuals from different backgrounds can reduce the likelihood of inbreeding depression and increase genetic diversity, it can also increase the risk of outbreeding depression and loss of local adaptations (Frankham et al. 2017).

The high value for parentage exclusion (QC = 0.9946) and the low genetic identity index (IC = 2.0362×10^−5^) indicate that the developed microsatellite loci have an excellent potential to exclude paternity and identify individuals. In addition, only four loci (Hha02, Hha03, Hha05 and Hha06; Supporting Table 3) are necessary to carry out these analyses with > 95% accuracy. This makes this panel of microsatellite loci practical for rapid characterization of individuals, since many individuals arrive in conservation centers with no metadata (Oliveira et al. 2022) and are then arbitrarily assumed to be related or not to other individuals from the *ex situ* population (Gonçalves da Silva et al. 2010; Nielsen et al. 2007).

Although the Harpy Eagle breeding in captivity has been successful, there is still a lack of genetic data from the *ex situ* population to support reproduction and conservation efforts. The captive breeding efforts for the Harpy Eagle in Brazil began in 1995 (Azeredo 2005), and since then, the number of individuals born in captivity has steadily increased. In 2020, Brazil had the largest *ex situ* population of Harpy Eagles, with 86 individuals born in the wild and 53 born in captivity (Oliveira et al. 2022), a number that has likely increased since then. However, the selection of breeding pairs is still based on behavioral compatibility between individuals, as well as information such as origin, age, and sex (Lerner et al. 2009; Oliveira et al. 2022; Watson et al. 2016).

Selecting breeding pairs based solely on sex identified through morphology can lead to incorrect choices, especially in species that do not exhibit clear sexual dimorphism. In the case of Harpy Eagles, there is no sexual dimorphism in feathers, but there is reversed sexual size dimorphism, where females are larger than males (Aguiar‐Silva et al. 2023; Banhos et al. 2023). Most zoos and breeders use body size as a criterion to determine sex, but this method can result in errors, as young females are often classified as males. In our study, for example, genetic analyses did not reveal any cases of misassigned sex in the individuals (Supporting Table 1).

However, individuals of the same sex can form behaviorally compatible pairs, resulting in the formation of numerous “pairs” that may never reproduce but are maintained as such for years. For instance, the Zoo Park da Montanha, the only zoo in Espírito Santo, maintained a pair for at least 8 years without any successful reproduction, until in 2019 we sexed the individuals and discovered that both were females. The downstream consequences of these misidentifications are clear, and minimally resulted in the inefficient use of scarce conservation resources. Therefore, there arises a necessity for a reliable, straightforward, and cost‐effective method for determining the sex of Harpy Eagles. This issue is addressed through molecular sex determination, first reported by Banhos et al. (2008) and further developed in this study (Supporting Information 1).

Endangered species generally have a limited number of breeding individuals in captivity, which increases the likelihood of matings between relatives (Francisco and Silveira 2013; Frankham et al. 2010). Therefore, it is critical that reliable data on relatedness of individuals in breeding programs exist. Our panel of microsatellite loci (Table 1) is able to provide this information with > 95% accuracy. Although one of the parent‐offspring relationships in the sampled family was misclassified as a full‐sibling relationship (HZ13‐HZ16, *r* = 0.7651, *R* = FS instead of PO), this categorical error is a consequence of the prior assumptions of the software. All kinship analysis software packages assume that the parents are unrelated, and therefore the expectation of genome sharing is 50% between parents and offspring (*r* = 0.5, *R* = PO) as well as between full‐siblings (*r* = 0.5, *R* = FS), with the difference that exactly one of the two alleles in the genotype of the parent must be observed in the offspring in a parent‐offspring relationship, whereas full‐siblings can share 0, 1, or 2 alleles per locus (Allendorf et al. 2013; Frankham et al. 2010). If parents are related this results in an elevated relationship coefficient (*r* > 0.5) and the statistical differentiation between PO and FS relationships becomes unreliable. Furthermore, it can be even more challenging to distinguish PO from FS when loci exhibit low diversity, as observed in this study, since a parent‐offspring pair may share alleles in a manner similar to two full‐siblings. Independent of the exact interpretation of kinships, in this and other similar cases the two individuals are very closely related, and their mating should be avoided.

We also observed that the relatedness coefficients were high for the known family, which may indicate inbreeding, especially since the parents of the offspring, HZ12 and HZ13, initially presented a relationship coefficient of *r* = 0.5. However, their relationship was classified as ambiguous and remained inconclusive even after hypothesis testing (Supporting Table 5). Therefore, when relatedness inference does not rule out the possibility of a genetic relationship between individuals, this type of pairing should be avoided whenever other options are available. It is essential to exercise caution when selecting reproductive pairs by evaluating not only the initial relationship coefficients but also their confidence intervals and hypothesis test results, to avoid pairing individuals with any level of relatedness.

The new set of microsatellite markers revealed the existence of other individuals that, when paired, present a certain level of relatedness. Most of these individuals come from geographically adjacent but distant areas, including neighboring states such as Pará and Amapá, as well as neighboring municipalities within the same state, such as Santarém and Tucuruí within Pará. These associations suggest the occurrence of Harpy Eagle reproduction in these regions and regional dispersal of offspring. The Harpy Eagle has a high capacity to explore vast areas during their flight and may disperse over long distances (Naveda‐Rodríguez et al. 2022; Urios et al. 2017), and therefore related individuals may be found across large geographic distances. Thus, individuals of unknown precedence may also be related to each other and to individuals of known precedence. Due to the conservation status of the Harpy Eagle, individuals of unknown precedence cannot simply be excluded from *ex situ* breeding programs, and at the same time, it cannot be assumed that they are unrelated to all other individuals. Once again, the importance of molecular data in guiding the formation of breeding pairs becomes evident, since one quarter of captive individuals are of unknown origin.

The Harpy Eagle have been shown to be geographically structured in three main groups: northern Amazon, southern Amazon, and the Atlantic Forest, with greater genetic similarity between the southern Amazon and Atlantic Forest groups (Banhos et al. 2016). The genetic diversity of the Harpy Eagle has also declined over the last 100 years, and it continues to decline (Banhos et al. 2016). Furthermore, the IUCN estimates that the Harpy Eagle population may decline by up to 50% over the next three generations (BirdLife International 2021). Therefore, it is essential to manage the captive population to avoid inbreeding and preserve genetic diversity. In addition, it is also necessary to maintain the natural genetic structure of the species, as it can directly reflect local adaptations (Frankham et al. 2017). This management is critical, as one of the ultimate goals of *ex situ* breeding and conservation programs is to produce individuals suitable for reintroduction into the wild (IUCN/SSC 2014). The microsatellite markers developed in this study can be used both to track genetic diversity over time, and to assign the likely origin of individuals to one of these three main groups.

In conclusion, we have shown that the microsatellite molecular markers together with the sex markers developed by Banhos et al. (2008) are effective molecular tools for filling gaps present in individual records and for managing the *ex situ* population. Therefore, we suggest that the entire *ex situ* population of the Harpy Eagle be screened using the loci developed in this study (Table 1) and the sex markers (Banhos et al. 2008), and that this information is entered into the record of each individual. The record of each individual should include behavioral information, geographic origin, and how they arrived at the institution, as well as any other relevant metadata. A more complete record for each individual will result in better informed *ex situ* conservation and management decisions, maximizing the efficiency and positive outcomes of these strategies while at the same time minimizing their cost.

## Ethics Statement

This study was conducted using blood samples collected by licensed veterinarians during routine veterinary procedures carried out at the zoological parks that provided material for genetic analyses. Since we did not directly collect the samples, handle the animals, or perform any animal experimentation, approval from the institutional animal ethics committee (UFAM) was not required for conducting genetic analyses. The transport of samples from the partner zoological parks to UFAM was done under the Brazilian Authorization and Information System on Biodiversity (SISBIO), a platform managed by Chico Mendes Institute for Biodiversity Conservation (ICMBio) for permitting research involving biodiversity in Brazil, license no. 31457‐6. This scientific disclosure was registered under no. AC7C51E in the National System for the Management of Genetic Heritage and Associated Traditional Knowledge (SISGEN), as required by Law no. 13,123/2015 and Decree no. 8,772 of May 11, 2016, in Brazil.

## Conflicts of Interest

The authors declare no conflicts of interest.

## Supporting information

Supplementary Material 1_18082025.

Supplementary Material 2_18082025.

Supplementary Tables S1‐S5_18082025.

## Data Availability

The data that support the findings of this study are available from the corresponding author upon reasonable request.
