# Supplementary material for: Development of Microsatellite Markers for Ex Situ Management of the Harpy Eagle Using Next Generation Sequencing
Source: Zoo Biol. 2025 Oct 9;45(2):97–108. doi: 10.1002/zoo.70030 (PMC13051753; doi:10.1002/zoo.70030)
Supplement: Supplementary file 1 — Supplementary Material 1_18082025. [file ZOO-45-97-s003.pdf]

Sex determination markers in *Harpia harpyja*

Protocol: Banhos et al. (2008)

1. **Primers used:**

Primers	5' - 3' primer sequence	Author
NP	GAGAAACTGTGCAAAACAG	Ito et al. (2003)
CHD1Wr	GCTGATCTGGTTTCAGATTAA	Banhos et al. (2008)
CHD1Zr	AGTCACTATCAGATCCAGAG	Banhos et al. (2008)

2. **Preparation of the PCR reaction mix:**

Component	Volume (µL)	Concentration
DNA	1	~20 ng
NP	2.5	2 µM
CHD1Wr	1.3	2 µM
CHD1Zr	1.3	2 µM
10X PCR Buffer	2.5	200 mM Tris-KCl, pH 8.5
$MgCl_2$	2.5	25 mM
dNTP	2.5	10 mM
Taq DNA polymerase	0.2	5 units/mL
Deionized water	11.8	
**Total reaction volume**	25	

3. **Thermocycling parameters:**

PCR Step	Temperature	Time	Cycle
Initial Denaturation	93ºC	1 min	
Denaturation<br>Primer Annealing<br>Extension	93ºC<br>52ºC<br>68ºC	10 sec<br>35 sec<br>30 sec	35
Final Extension	68ºC	7 min	

4. **Analyze the results of your PCR reaction via gel electrophoresis on 2% or 3% (p/v).**

5. **Example of the result:** Agarose gel visualization of a sex identification test. In total, we used thirteen samples: two as controls from known individuals (one female and one male); four are females (♀; ZW; two bands); seven are males (♂; ZZ; one band).

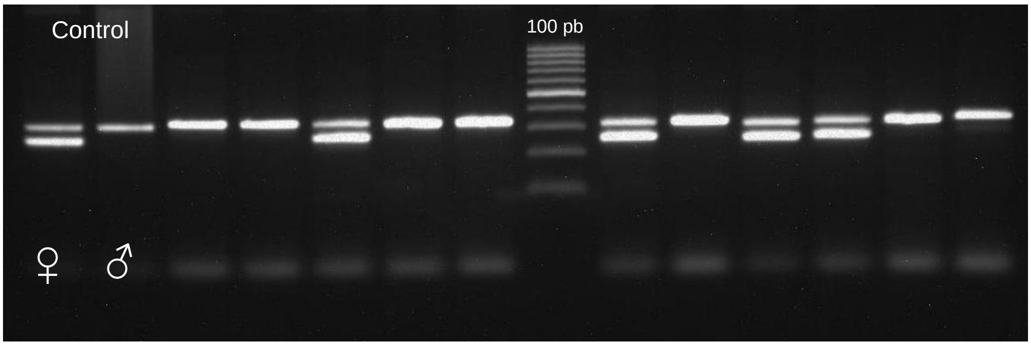

References:

Banhos, A., Hrbek, T., Gravena, W., Sanaiotti, T., & Farias, I. P. (2008). Genomic resources for the conservation and management of the Harpy Eagle (*Harpia harpyja*, Falconiformes, Accipitridae). *Genetics and Molecular Biology*, *31*(1), 146–154. https://doi.org/10.1590/S1415-47572008000100025

Ito, H., Sudo-Yamaji, A., Abe, M., Murase, T., & Tsubota, T. (2003). Sex Identification by Alternative Polymerase Chain Reaction Methods in Falconiformes. *Zoological Science*, *20*(3), 339–344. https://doi.org/10.2108/zsj.20.339
