# Supplementary material for: Development of Microsatellite Markers for Ex Situ Management of the Harpy Eagle Using Next Generation Sequencing
Source: Zoo Biol. 2025 Oct 9;45(2):97–108. doi: 10.1002/zoo.70030 (PMC13051753; doi:10.1002/zoo.70030)
Supplement: Supplementary file 2 — Supplementary Material 2_18082025. [file ZOO-45-97-s001.pdf]

Genotyping of new microsatellites for *Harpia harpyja*

Protocol: Kaizer et al. (2025) - this study.

1. **Primers used:**

Primers	Repetition *Motif*	5' - 3' primer sequence	Size (bp)	T_a (°C)
Hha01	$(AC)_{10}$	F- CTCCTGTGTTCCCATCTGCT<br>R- GGGTGCGAGACAGTTCCTC	158	59
Hha02	$(AC)_{10}$	F- TCTGTTGGAGTTTCCGGAGC<br>R- CCGGGATGCATCTCCTTTGT	192	59
Hha03	$(AG)_{14}$	F- ATGGCAAGTCGCTAGAACGG<br>R- GGCTGCTATTCCACTTGCCA	204	57
Hha04	$(AC)_{12}$	F- GTGGAAGGGAGTTAGCGTGG<br>R- ATGGCTCTGTCTAACCTGCG	125	59
Hha05	$(AGC)_{11}$	F- ACAAGGTCACATCTTCCCGC<br>R- AAACTGTTCTGCCTCGCTGA	179	59
Hha06	$(AG)_{12}$	F- ACCAGGCTGTAAGGGTTGAAG<br>R- TGGACAGATGTGGAGTGTCG	121	59
Hha08	$(AC)_{10}$	F- CATCCGTAGCCATGCACAGA<br>R- CTTACCTGGGTGTCAGCACA	155	57
Hha09	$(AC)_{12}$	F- ACCCAAGCTCTTCCTGGATG<br>R- TCAAAGCGTATGAAGCCAGGA	198	57
Hha12	$(AG)_{11}$	F- TCCTCCTTGGAAGCAGCAAG<br>R- AGCTCAGTTTCTGGAGTCAGG	120	57
Hha18	$(AC)_{11}$	F- GCCCACAGAAACGATACAGC<br>R- ATTAGCTGCTCGCAGACAAA	150	57

2. **Preparation of the PCR reaction mix separately for each primer:**

Component	Volume (µL)	Concentration
DNA	1	~20 ng
Primer Forward	1	0.2 pM
Primer Reverse	1	0.2 pM
10X PCR Buffer with $(NH_4)_2SO_4$	1	75 mM Tris-HCl pH 8.8 at 25 °C, 20 mM $(NH_4)_2SO_4$
$MgCl_2$	1	2.5 mM
dNTP	1	1 mM
BSA	0.5	2 mg/mL
Taq DNA polymerase	0.3	0.1 U
Nuclease-Free ddH_2O	3.2	
**Total reaction volume**	10	

3. Thermocycling parameters:

PCR Step	Temperature	Time	Cycle
Initial Denaturation	94ºC	1 min	
Denaturation	94ºC	30 sec	
Primer Annealing	-- ºC	30 sec	30
Extension	68ºC	40 sec	
Final Extension	72ºC	5 min	

Note: (--) the primer pairing temperature will vary according to the table in the first topic.

4. Analyze the results of your PCR reaction via gel electrophoresis on 1% (p/v).

5. Individual pool creation:

After all samples have been amplified for each primer, we form the individual pool. To do this, we transfer 3 µl of each sample/PCR product to a single 0.2 ml Eppendorf microtube or to the 96-well plate (depending on the number of samples being used).

6. Purification of the individual pool:

We used the magnetic beads prepared according to the protocol BOMB #4.2 (Oberacker et al., 2019) and following the steps below:

- Add 0.8 µl of beads for each 1 µl of individual pool.
- Vortex quickly and spin in a centrifuge for 3 seconds.
- Incubate for 5 minutes at room temperature.
- Place the tube on the magnetic rack and incubate for another 5 minutes at room temperature.
- Remove the supernatant and centrifuge together with the magnetic rack at 100 ref for 30 seconds.
- Wash the beads by adding 150 µl of ice-cold 80% alcohol.
- Remove the supernatant AGAIN and centrifuge together with the magnetic rack at 100 ref for 30 seconds.
- Wash the beads AGAIN by adding 150 µl of ice-cold 80% alcohol.
- With the tubes still on the magnetic rack, dry them in the oven for 10 minutes.
- Wait for it to cool and resuspend in water twice the initial volume.
- Homogenize, spin in a centrifuge for 3 seconds and incubate in the refrigerator for 5 minutes.
- Afterwards, store in the freezer.

7. Analyze the results of your purification via gel electrophoresis on 1% (p/v).

8. Preparation of adapters A and P1:

The mix preparation for adapters A and P1 is carried out separately, as show in the table below:

Component	Volume (µL)	Volume (µL)	Concentration
Primer A	0.8	-	0.2 pM
Primer P	-	0.8	0.2 pM
10X PCR Buffer with $(NH_4)_2SO_4$	0.5	0.5	75 mM Tris-HCl pH 8.8 at 25 °C, 20 mM $(NH_4)_2SO_4$
$MgCl_2$	0.5	0.5	2.5 mM
dNTP	0.5	0.5	1 mM
BSA	0.25	0.25	2 mg/mL
Taq DNA polymerase	0.15	0.15	0.1 U
Nuclease-Free ddH_2O	1.8	1.8	
**Total reaction volume**	4.5	4.5	

9. Indexing reaction of adapters A and P1:

Add 4.5 µL of the previously prepared A adapter and P1 and 1 µL of the purified individual pool into a tube, totaling 10 to perform the indexing reaction.

10. Thermocycling parameters:

PCR Step	Temperature	Time	Cycle
Initial Denaturation	94ºC	1 min	
Denaturation<br>Primer Annealing<br>Extension	94ºC<br>48ºC<br>68ºC	30 sec<br>30 sec<br>40 sec	15
Final Extension	72ºC	7 min	

11. Analyze the results of indexing via gel electrophoresis on 1% (p/v)

12. Lybrary creation:

After all individuals were encoded using adapters A and P1, we removed 3 µL from each individual pool and transferred it to a 1.5 ml eppendorf tube, forming the total library.

13. Purification of the lybrary similar to topic 6.

14. Next generation sequencing.

15. Demultiplexing of individuals:

We used the fqgrep tool (github.com/indraniel/fqgrep), searching for combinations of adapters A and P1 and storing the reads in a fastq file for each individual.

References:

Oberacker, P., Stepper, P., Bond, D. M., Höhn, S., Focken, J., Meyer, V., Schelle, L., Sugrue, V. J., Jeunen, G.-J., Moser, T., Hore, S. R., Von-Meyenn, F., Hipp, K., Hore, T. A., & Jurkowski, T. P. (2019). Bio-On-Magnetic-Beads (BOMB): Open platform for high-throughput nucleic acid extraction and manipulation. *PLOS Biology*, *17*(1), e3000107. https://doi.org/10.1371/journal.pbio.3000107
